# Determinants of Menstrual Hygiene Management Practices among Schoolgirls: A Cross-Sectional Study in the Savannah Region of Ghana

**DOI:** 10.1155/2022/7007117

**Published:** 2022-08-08

**Authors:** Mubarick Nungbaso Asumah, Abdulai Abubakari, Gifty Apiung Aninanya

**Affiliations:** ^1^Ghana Health Service, Kintampo Municipal Hospital, P.O. Box 192, Kintampo, Bono East, Ghana; ^2^Department of Global and International Health, School of Public Health, University for Development Studies, P.O. Box TL1350, Tamale, Northern Region, Ghana; ^3^Department of Health Services Policy, Planning, Management and Economics, School of Public Health, University for Development Studies, P.O. Box TL1350, Tamale, Northern Region, Ghana

## Abstract

**Introduction:**

Menstruation is crucial in the reproductive lives of all women. The advent of menses in most settings is accompanied by physical and psychological health, religious, social, and cultural implications. The research intends to identify determinants of menstrual hygiene management (MHM) practices among adolescent girls in Junior High Schools in the West Gonja Municipality of the Savannah Region of Ghana.

**Methods:**

The study employed an analytical cross-sectional design with 430 adolescent schoolgirls selected through multistage sampling techniques. A structured questionnaire was used to collect data and analyzed using STATA version 14. A logistic regression model was run to determine the predictors of MHM practices.

**Results:**

The study discovered that 63.7% of the girls had sufficient knowledge of menstruation and menstrual hygiene. Almost all girls (97%) used some form of absorbent materials during menses, with over half of these girls (58.6%) using commercial sanitary pads, 30.5% using cloth, 3.7% using cotton, and 4.2% using tissue papers with 3.0% reported not using any absorbent material. Only 44.4% reported reusing their absorbent materials. Out of which, the majority (88.5%) of the schoolgirls cleaned their reusable absorbent material using soap and water with 77.5% drying absorbent materials in the sun. Overall, 84.9% practiced good MHM. Type of school [Adjusted Odds Ratio (AOR) =6.0; 95% Confidence Interval (CI) (2.64-13.59)], pocket money [AOR =2.5; 95% CI (1.27-4.86)], and residence [AOR =2.8 95% CI (1.55-5.18)] were the most significant determinants of menstrual hygiene management practice.

**Conclusion:**

About two-thirds of the schoolgirls are knowledgeable in menstrual hygiene but access to management materials is problematic whereas approximately half of the girls have access to sanitary pads and the rest resort to the use of cloth and cotton. Pocket money and residential status were the most important predictors of the menstrual hygiene management. The government initiative to provide schoolgirls with sanitary pads could go a long way to improve menstrual hygiene management if implemented across all schools in Ghana, particularly in rural areas.

## 1. Introduction

Menstruation is a natural process that gives rise to several fluctuations in a female life that causes emotional and psychological instability [1]. It represents a significant breakthrough in a woman's life as it symbolizes the start of reproductive capacity, which is a desire of most women [2]. Adolescence is a chapter in an individual's life, where an individual changes from juvenile to mature life in which pubertal growth and sexual development take place field [3].

Globally, females who are of their reproductive age are over 50% of the population of the sex group [4]. The Joint Monitoring Program (JMP) of WHO and UNICEF defined menstrual hygiene management (MHM) as “Women and adolescent girls using a hygienic menstrual management material to collect blood that can be changed in privacy as often as necessary for the duration of the menstrual period, using soap and water for washing the body as required, and having access to facilities to dispose of used menstrual management materials” [5].

Poor MHM influences girls negatively globally, especially those in the developing world [6, 7]. It usually leads to ailments, including reproductive tract infection, and urinary tract infections field [8] as well as posing a negative effect on the environment as it generates a waste mess if no appropriate disposal approaches put in place [9]. That said, the importance of good menstrual hygiene management cannot be underestimated because it increases confidence and helps to improve the physical, mental health, education, and self-esteem of schoolgirls [1].

Several factors affect the hygiene being practiced by adolescent girls during menses. MHM practice is prejudiced by a lot of indicators including adolescents' understanding of menstruation, availability of suitable facilities, and environment (be it social or cultural) to accomplish menstruation hygienically and with pride [1, 10, 11]. Also, a study has shown that in most developing countries, more than 50% of girls practice poor menstrual hygiene management, with the majority of them coming from rural areas [12]. For instance, in Ethiopia, almost all adolescents (96.9%) were engaged in very poor menstrual management [13]. Most women and girls cannot afford the cost of a sanitary pad in Zambia and use rags to absorb menstrual flow due to poverty [4]. In poorly resourced countries, access to sanitary materials and suitable sanitary facilities are very few thereby posturing a massive challenge to the menstruating girls [14].

It is common knowledge that menstruating women in Northern Ghana are often unable to cook; they do not sleep with their husbands [15]. All these misconceptions affect gender equality, access to education, and others. Presently, tremendous contributions from academia, development actors, and all stakeholders to curb the difficulties of the adolescent girl (especially the schoolgirl) in underdeveloped countries [6] are remarkable. Despite the apparent depth of literature on MHM among female students in Ghana, various studies have reported that the girls in their menses face various challenges, including embarrassment, distress, and misperception, managing issues of menstruation with deficient information, absence of social support, scarcity of water, and hygienic and waste disposal amenities in school environments field [16–18].

The adolescent population according to the Ghana Demographic and Health Survey [19] is about 22% of Ghana's population, of which girls are the majority. The West Gonja Municipality has at least 10,518 adolescents representing 25.5% of the area population [20]. Although there is no information on the hygiene facilities in schools in Savannah Region, other studies conducted in Northern Ghana have shown that there is inadequate hygiene infrastructure in basic schools [21, 22]. Together with the misconceptions mentioned earlier, in the study setting, menstruating girls are not supposed to share a common bathroom, they are not supposed to welcome a male into the house, etc. These misconceptions may have some implications on the MHM practices in the municipality. However, there is limited literature on menstrual hygiene among schoolgirls in the newly created Savannah Region in Northern Ghana. This study was therefore designed to identify the determinants of menstrual hygiene management practices among schoolgirls in the West Gonja Municipality of the Savannah Region of Ghana. This could lead to the design of appropriate interventions and strategies at local and national levels to provide the necessary facilities, education, and utilities for the good practices of menstrual hygiene among these schoolgirls. Furthermore, the problem of MHM is multifaceted, necessitating varied methods to tackle it. As a result, the research will give data to the Ministries of Health and Education at the regional level for planning and policy formation.

## 2. Materials and Methods

### 2.1. Study Design and Setting

An analytical cross-sectional study design was carried out in the West Gonja Municipality. Damongo is the administrative capital of West Gonja Municipality. It was created in 2004 by a new law (L.I.1775) [20]. It also bonds to the south with Central Gonja District, Bole and Sawla-Tuna-Kalba Districts to the west, Wa East District to the north-west and North Gonja District to the east. The capital of the Municipal also doubled as the Capital of the newly created Savannah Region. The Municipal has eighty-five (85) basic and second cycle institutions: Thirty-four (34) are kindergarten and nursery, 33 primary schools, 17 Junior High Schools and only three (3) Senior High Schools [20].

### 2.2. Study Population

The study population consisted of adolescent schoolgirls who have had their menses and lived in the West Gonja Municipality.

### 2.3. Sample Size Determination and Sampling Procedure

The sample size was computed using the Snedecor and Cochran [23] formula for a point estimate sample; *N* = sample size, *z* = *z*-score of a 95% confidence level equivalent to 1.96, and *p* = proportion of MHM practice among girls was projected as 50%. This is because 50 percent coverage yields the largest sample size. *q* = estimated proportion of teen girls who do not practice good menstrual hygiene management (1 − *p* = 0.5), and *m* = margin of error of the study =5% =0.05 in this study, so that whether the actual prevalence was less than or more than 50%, the needed sample size would have been covered regardless. With a non-response rate of 10%, a total of 430 girls were recruited.

The study used multistage sampling. The West Gonja Education directorate provided the circuits within the Municipality (6 circuits) and the number of schools under each of them. Therefore, each circuit was considered a stratum and four out of the six circuits were selected using a simple lottery. For each stratum, three (3) schools were chosen using simple random sampling. For each chosen school, the list of students who fall within the inclusion criteria was obtained (i.e., sample frame), so using systematic sampling, a total of thirty-six (36) students were chosen from each school, thus making the sample unit.

### 2.4. Data Collection Tools and Techniques

A structured questionnaire was used to gather data from the adolescent girls who have experienced at least one menses. Before data collection, the research assistant clarified the objectives of the study and obtained verbal and written consent. The questionnaire was adapted from existing literature [24–27] and modified to suit the study setting and participants. A structured questionnaire was used because the majority of the respondents could read and write in the English language and face to face data collection techniques for those respondents who needed to be supported with the understanding of the questionnaire. The questionnaire administration was supported by four (4) female undergraduate nursing students. These enumerators were trained on the data collections tools and study protocol. Though self-administered, for those who could not read and write well enough, the questionnaire was appropriately translated into the local dialect (Gonja or Kamara) for easy assimilation to solicit the appropriate response from the chosen respondents. Data collection started in January 2020 and ended in March 2021. The questionnaire was structured in three sections in line with the objectives of this study.

Section A solicited information on the socio-demographic characteristics of the girls. This included age, sex, name of the school, occupation, residence type, and educational levels of parents.

Section B was on knowledge of menstruation and menstrual hygiene. Some of the variables included the following: nature and causes of menstruation, the channel of information on menstruation, knowledge of menstrual hygiene, sources of menstrual blood, and menstrual hygiene being unhygienic and foul smell during menses.

Section C was on the practices of menstrual hygiene among adolescent girls. This section focused on asking hygiene-related questions during menses which included but were not limited to the following; type of absorbent materials used, how it is washed and cleaned, how it is dried and stored, bathing during menses, and medication use during menses.

A school within the Municipality was chosen for pretesting to further ensure the reliability and accuracy of the tool. Questionnaires were examined at the end of each day's work to ensure that incomplete questionnaires were removed. Adequate plans were put in place to ensure that data collected were entered and clean using data analysis software to ensure higher accuracy.

### 2.5. Data Analysis and Presentation of Results

Quantitative data was coded and analyzed using Stata version 14. Analysis was done using expressive and inferential statistics and was presented using tables and a figure.

Consistent with Upashe, Tekela, and Mekonnen [24], the calculation of the knowledge score on menstruation was done using seven (7) specific questions (what is menstruation, cause of menstruation, source of menstrual bleeding, information on menstruation before menarche, foul smell during menses, knowledge on menstrual hygiene, menses unhygienic). For each of the aforementioned questions, a correct answer scored one (1) point while wrong and do not know answers scored no (0) points. Thereafter, the mean (4.82 ± 1.05) was used as a cut-off point to categorize the knowledge score into sufficient and insufficient. Thus, those who obtained 5 to 7 correct responses were said to have had sufficient knowledge of menstruation and menstrual hygiene whereas insufficient knowledge of menstruation and menstrual hygiene were those who scored below 5 (0-4 correct responses).

The respondents self-reported their MHM practice experiences with the aid of a structured questionnaire. Respondents were asked whether they used absorbent materials during menses. Those who answered “yes” were further asked about the materials used to catch menstrual blood. To know the cleanliness of the absorbent materials, a question was asked: “do you reuse your absorbent materials.” Respondents who answered in the affirmative were questioned on the washing and drying of the absorbent materials.

Also, the frequency of change of absorbent materials within 24 hours was assessed as well as the place for disposing of used disposable materials. Respondents were asked if they bathed during the menstrual period, and the number of times they bathed in a day. To assess how the genitals were kept during menses, a question was asked: “do you clean your genitals during menses.” Those who answered “yes” were asked about the “material use to clean the genitals.”

On the MHM practice criteria, various studies [24, 27] have presented different measures for this outcome. However, concerning the Joint Monitoring Program (JMP) of WHO and UNICEF definition of menstrual hygiene management (MHM) [5] and the observation by Sumpter and Torondel [28] in a systematic study, there is no reliable standard in the measure of “good” and “bad” MHM; this study categorized the MHM practices into “good” or “poor” MHM practices. More relaxed parameters were employed to further categorize the MHM practice as “good” or “poor.” These criteria have been used elsewhere and have proven to be a reliable measure for the MHM practices [29]. To do this, those who were reported to be using absorbent materials during menses were said to have good MHM practices. Also, those who used sanitary pads and new clothes were said to have good MHM practices while those who use tissue paper, old homemade pads, and old cloth were said to have poor MHM practices. To ensure the cleanliness of absorbent materials, those who were using reusable absorbent materials were further questioned on the washing and drying of these materials. Washing absorbent materials with soap also helps in reducing infections. Leaving reusable materials unwashed has been proven to promote microbial survival, and wearing them wet has long been deemed unsanitary, with some evidence of increased infection risk and discomfort [30, 31]. Drying techniques are also crucial, as UV exposure from sunshine has been shown to have a microbicidal impacted [30]. From above, respondents who washed their absorbent materials with soap and reported drying them in the sunlight were categorized as having good MHM practices. On the other hand, those who did not wash or use only water to wash absorbent materials and those who dry their absorbent materials in the room and any other place aside from sunlight were said to have poor MHM practices. On the frequency of changing absorbent material, two (2) or more times were considered a good MHM practice. This is because House Mahon and Cavill [31] recommended changing absorbent materials every two (2) to six (6) hours depending on the menstrual flow. Respondents who bathed during menses and cleaning of the genitalia with only water were all regarded as having good MHM practices.

Although “place for disposable of absorbent material,” “frequency of bathing,” and “the material used in cleaning genital” during menses were reported under practice, there was no consistent literature linking these variables to good MHM practice or otherwise. Hence, these variables were not included in the overall MHM practice.

There were no suitable questions in the current study to capture the capacity to change absorbent materials privately, which might have increased the MHM practice calculation. Supplemental Table s1 is the knowledge and practice scoring scale.

Respondents were needed to fulfill all criteria listed above to be classified as having good MHM practices, while those who did not meet all relaxed requirements were classified as having poor MHM practices. Hennegan et al. [29] employed this criterion and found it to be a more accurate metric for MHM practices.

Bivariate analysis was used to detect association among study variables and a *p*-value lower than 0.05 was set as being statistically significant. A logistic regression model was estimated to determine the determinants of menstrual hygiene practices.

On the determinants of MHM, all variables with *p* values less than 0.2 were entered into the logistic regression model. The *p*-value of 0.2 was selected because of its closeness to zero (0) thus would reveal the most desired effect and improved multivariate model (binary logistic model). To assess the determinants of menstrual hygiene management practice, the study used a binary dependent model called the logistic regression model. This model uses the Odds Ratio estimator to predict the outcome of each independent variable on the dependent variable. The use of the model is also justified based on the fact that MHM practice can be modelled as a dichotomous decision, thus to practice “Good” or “Poor” MHM. Hence, this model denotes that good MHM practice is given a value of 1 and 0, if otherwise (poor MHM practice).

### 2.6. Ethical Approval

Ethical clearance was obtained from the Committee on Human Research, Publications & Ethics (CHRPE) with reference CHRPE/AP/199/20. The Director of Education for West Gonja Municipality and the heads of chosen schools gave their approval. Before participating in this survey, each respondent gave their permission. After receiving complete information about the study, participants gave signed informed consent. However, for participants under 16 years, consent was obtained from parents or guardians. Subjects who refused to provide their consent were not allowed to participate in the study. Participants were informed that participation in the study was entirely optional and that they may opt out at any moment during the procedure if they so desired. There were no monetary rewards for those who took part. All participants were informed that the study's findings will be made available to the general public. Finally, this study was conducted by the principles of the Declarations of Helsinki.

## 3. Results

### 3.1. Socio-Demographic Characteristics

This study was carried out among 430 adolescent schoolgirls in the West Gonja Municipality. The average age of the girls was 15.10 ± 1.50. The majority (68.4%) of the girls are between the ages of 15 and 19 years, and most of the girls (40.9%) were in form two (2) with the majority of the girls being Gonjas (52.3%). The majority of the girls were Muslims (76%), were in public schools (91.6%), stayed with both parents (56.7%), were residents in the rural settings (52.0%), and only 38.4% get pocket money (Table 1).

### 3.2. Knowledge of Schoolgirls on Menstruation and Menstrual Hygiene

**Figure 1:**
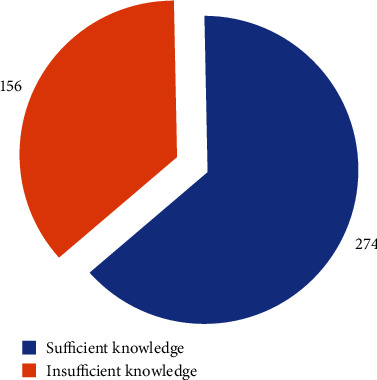
Overall knowledge of menstruation and menstrual hygiene.

The majority of the respondents (63.7%) had sufficient knowledge of menstruation and menstrual hygiene compared to 36.3% who did not know about menstruation and menstrual hygiene (Figure 1).

### 3.3. Menstrual Hygiene Management (MHM) Practices among Adolescent Schoolgirls

The study found that the majority (97%) of the girls use absorbent material during their period, with more than half of these girls (58.6.%) using commercial sanitary pads, 30.5% using cloth, 3.7% using cotton, and 4.2% using tissue papers while 3.0% reported not using any absorbent material. However, only 44.4% reported reusing their absorbent materials. Out of those reusing absorbent materials, the majority (91.1%) cleaned their reusable absorbent material using soap and water with 80.1% drying absorbent materials in the sun. Most of the girls (49.8%) changed their absorbent materials twice a day. A greater proportion (90.9%) of the girls wrap their absorbent materials before disposing of them. All but 1.4% of the girls bathed during their periods with most (46.0%) bathing twice during their period. On the disposal of used absorbent materials, the majority (65.4%) dispose of them in the toilet, 19.3% in the drains, and 12.3% in the open fields. Overall, the majority (84.9%) of the girls observed good menstrual hygiene management practices (Table 2).

### 3.4. Determinants of Menstrual Hygiene Management

The study discovered that girls in public schools were 6.0 times more likely to practice good menstrual hygiene management (AOR; 6.0, 95% CI; 2.64-13.59) as against those in private schools. Adolescent girls with pocket money were 2.5 more likely to practice good menstrual hygiene management as compared to adolescent girls who had no pocket money (AOR; 2.5; 95% CI; 1.27-4.86). Girls who reside in the urban areas were 2.8 times more likely to practice good menstrual hygiene management (AOR; 2.8 95% CI; 1.55-5.18) compared to those who reside in rural areas (Table 3).

Ref^∗^: reference, *p* < 0.05^∗^, *p* < 0.01^∗∗^, *p* < 0.001^∗∗∗^; COR: Crude Odds Ratio; AOR: Adjusted Odds Ratio; CI: Confidence Interval.

## 4. Discussions

This study aims at identifying the determinants of menstrual hygiene management practices among schoolgirls in the West Gonja Municipality of the Savannah Region of Ghana. The study observed that 63.7% of the girls had sufficient knowledge of menstruation and menstrual hygiene. This is closer to what was found in Chitwan, Nepal (66.8%) [27], and in western Ethiopia (60.9%) [24]. However, the current findings are higher than the 52.5% found in India [26] and 51.6% found in Kenya [25]. The higher levels of knowledge on menstruation and menstrual hygiene show that the ongoing efforts to disseminate information on menstruation in basic schools in Ghana through the school health program are achieving considerable outcomes.

The study showed that commercial sanitary pads (58.6%) and cloth (30.5%) were commonly used among participants. The findings of the current study on the usage of sanitary pads were lower than that of 98.6% in Egypt [32], 93.8% in Nepal [27], and 67.2% % in Nigeria [33]. This current finding is however higher than that of 47.2% in rural Ghana [34]. In the present study, the study participants were only schoolgirls whereas the one conducted in rural Ghana (33) covered all categories of adolescent girls (those in school and out of school) which could be the reason for the differences in the data presented above.

The second most common absorbent material used was cloth (30.5%). These findings are lower than 57% in Northern Ghana [35] and similar to 26.9% in Nigeria [36]. Contrary to the above, studies conducted in Bangladesh by Anee and colleagues [37], and India by Pradeepkumar et al. [26], revealed that the majority of the respondents used a cloth to absorb menstrual blood during their menses. This was supported by Wiesemann and Adam [38], who opined that sanitary pads handle different densities of menses under a variety of conditions and activities, are easy to use, comfortable, and easy to dispose of. The socio-economic status and socio-cultural factors (myths, taboos, parent's occupation, educational level, etc.) could be linked to the differences in the findings.

Cleaning of reusable absorbent materials is crucial to the effective practice of the hygiene management [9]. This study revealed that the majority (91.1%) of respondents who used reusable materials washed them with water and soap. This is higher than a study in rural Uganda [39] which showed that 73.4% of the girls washed their cloth with water and soap and 52.9% as reported in Ethiopia [24]. On the contrary, in Mumbai, India, most respondents washed their reusable absorbent materials with only water [40]. This discrepancy could be explained using the economic status of the parent. The availability of soap for washing absorbent materials may be absent hence compelling them to wash absorbent materials with only water.

Nearly 80.0% of girls who use reusable absorbent material dry them in the sun. This is higher than similar studies in western Ethiopia [24] which reported that 45.2% of the girls dried their reusable material in the sun. The drying of these materials in the sun is considered the best practice since the sunlight serves as a natural sterilizer to cleanse the material of any bacteria that could harm the health of the user [30]. In areas where adequate sunlight is not available, ironing of the material after they are dried serves the same purpose as mentioned by Sommer et al. [6]. Though drying the absorbent material in the sun is crucial, not all those using reusable absorbent material can dry it in the sun as a result of shyness and lack of private places for drying. This may compel some to dry their absorbent materials in the room. Besides, Hennegan et al. [39] reported that some girls in a boarding school in Uganda were shy to wash their used cloth in public; they washed in the night and dried them in the dormitory. It is therefore very necessary to empower adolescent girls and women in general with the right information on menstrual hygiene and its management as this might aid in changing the mindsets or perceptions regarding MHM practices.

Almost all respondents reported taking regular baths during their menstrual periods. This is similar to Mohammed et al. [34], who reported a 94% bath in rural Ghana during menses. Though there are no clear-cut references on the frequency of baths during menses, it is very essential to have suitable hygiene and wash the body and hair each day, especially during the menstrual period because it makes girls feel more confident. Findings of the current study showed that over 90.0%of girls bathed more than once during menstruation than in similar studies in Chitwan, Nepal, which revealed 64.2% of study participants bathed more than once [27], and in western Ethiopia, 67.8% of the girls were bathing more than once [24]. Contrary to the above, Michael et al. [41], in a study conducted in Quetta, Pakistan, revealed that more than half (58.2%) of the study participants did not bathe at all during their menses. This discrepancy could be attributed to the availability and accessibility of water. Also, there may be some cultural underpinnings in the different geographical areas.

It was observed that the majority of the girls purposely clean their genitalia during their period. This purposeful genitalia cleansing was undertaken on multiple occasions with some girls washing at least three times daily during their period. The current finding is seen to be higher in other studies in other locations. For instance, in Kenya, Mathenge and Midigo [42] reported that about 84% of study participants washed their genitalia during menses. As revealed in this study, a higher proportion of schoolgirls used soap and water in washing their genitalia. This is similar to a study where slightly higher than 54.4% reportedly used soap and water to clean their genitalia in rural Puducherry [43]. Consequently, cleaning genitalia with soap and water amounts to douching which can cause the overgrowth of harmful bacteria in the vulva thereby causing illness [44]. The practice of washing the genitals with soap and water needs to be discouraged. On the other hand, only a few participants (26.7%) of the schoolgirls use water only in washing their genitals during menses. This finding of the current study is higher than 4.6% who reported to be washing their genitals with water in Nigeria [45]. This is considered the best way of cleaning the genitalia provided the hands are clean to prevent further introduction of harmful bacteria into the vulva. As such, the girls should be encouraged to wash their hands properly before and then use clean water to wash their genitals.

The majority of the respondents (84.9%) practiced good menstrual hygiene management. This finding is higher than studies carried out elsewhere in Ghana. For example, a study by Mohammed et al. [34] showed that little over half of the sampled girls had good menstrual hygiene management in rural communities; in Kenya, 64.3% of study participants had demonstrated good menstrual hygiene management practices [42], in Nepal 72.5% of the respondents practiced good MHM [27], and in western Ethiopia, 33.9% practiced good MHM [24]. The finding of this study is similar to a study in Persia, where 78.7% of respondents had good menstrual practice [46] and in Egypt, another study [32] showed that about 90.0% of hygienic practices of adolescent schoolgirls are towards menstruation.

The current study discovered that adolescent girls with pocket money were 2.5 times more likely to practice good menstrual hygiene management as compared to adolescent girls who had no pocket money. These findings are similar to a study in Ethiopia where students with no social support (no pocket money) were 64% less likely to practice proper menstrual hygiene as compared to their counterparts who had permanent pocket money [47]. Most schoolgirls were reported to stay with their parents. These parents who provide money for the upkeep of their girls also provided needed support including monetary support during menstruation to enable them effectively manage their menstrual experiences.

Adolescent girls in this study who reside in the urban areas were more likely to practice good menstrual hygiene than those in rural areas. This is related to a study by Mutunda [4] who observed that women who reside in villages and cottages and have poor families are unable to pay for commercial used sanitary pads; instead, these women use rags or cloth, often obtained from old clothes. These rags or cloths are usually washed without following the right procedures with insufficient and unsafe water and lacking soap and used repeatedly without drying under the sun. These practices could lead to urinary and reproductive infections [48].

Finally, the study revealed that girls in public schools were more likely to practice good menstrual hygiene management compared to their counterparts in private schools. This observation contradicts the finding of a study in Pakistan which showed that good menstrual hygiene management practices were 48.1% in private centers compared to 33.7% in government-owned schools [49]. The discrepancies could be attributed to the government's support of public schools in Ghana.

However, it is worth mentioning that the result as reported by the current study must be interpreted with caution as the study participants included only students in selected basic schools in the West Gonja Municipality, which were largely public facilities. These categories of adolescents are exposed to a lot of information and could not be generalized to all adolescent girls in the Municipality as the situation for those who are not in school may be different. Due to social desirability, reporting bias could not be ruled out but effort was made by the researchers to minimize the impact of reporting bias on the findings. Notwithstanding these limitations, the study provides insight into menstrual hygiene practices among adolescent girls in the Municipality.

## 5. Conclusion

About two-thirds of the schoolgirls are knowledgeable in menstrual hygiene but access to management materials is problematic whereas approximately half of the girls have access to sanitary pads and the rest resort to the use of cloth and cotton. Pocket money and residential status were the most important predictors of the menstrual hygiene management. The government initiative to provide schoolgirls with sanitary pads could go a long way to improve menstrual hygiene management if implemented across all schools in Ghana, particularly in rural areas.

## Figures and Tables

**Table 1:** Socio-demographics of the girls (*n* =430).

Variables	Categories	Frequency	Percentages
Age	<15 years	136	31.6
	≥15 years	294	68.4
	Mean ± SD		15.10 ± 1.50
Class	JHS 1	142	33.0
	JHS 2	176	40.9
	JHS 3	112	26.1
Ethnicity	Gonja	225	52.3
	Kamara	85	19.8
	Others	120	27.9
Religion	Islam	327	76.0
	Christianity	103	24.0
Category of school	Public	394	91.6
	Private	36	8.4
Residence	Rural	216	50.2
	Urban	214	49.8
Living with	Mother/father only	88	20.5
	Both parent	244	56.7
	Relatives	89	20.7
	Boarding	5	1.2
	Friends	4	0.9
Mother's occupation	Formal job	27	6.3
	Informal job	403	93.7
Pocket money	Yes	165	38.4
	No	265	61.6

**Table 2:** Menstrual hygiene management practices among adolescent schoolgirls (*n* =430).

Variables	Categories	Frequency	Percentages
Do you use absorbent materials	Yes	417	97.0
	No	13	3.0
Which absorbent materials do you use	Disposable sanitary pad	252	58.6
	Cloth	131	30.5
	Cotton	16	3.7
	Tissue paper	18	4.2
	Absorbent not used	13	3.0
Do you reuse absorbent materials	Yes	191	44.4
	No	239	52.6
	Absorbent not used	13	3.0
How to clean absorbent material (*n* =191)	Only water	17	8.9
	Soap and water	174	91.1
Where do your dry absorbent materials (*n* =191)	Inside the room	38	19.9
	Sunlight	153	80.1
Frequency of change of absorbent material	Absorbent not used	13	30.0
	Once	57	13.3
	Twice	214	49.8
	Three and more	146	34.0
Where do you dispose absorbent material	Drains	82	19.3
	Open field	53	12.3
	Toilet	281	65.4
	Absorbent not used	13	3.0
Do you bath during menses	Yes	424	98.6
	No	6	1.4
Frequency of bathing	Daily	19	4.4
	Twice daily	198	46.0
	Thrice daily	188	43.7
	More than 3 times	19	4.4
	Until the end of the menses	6	1.4
Genitalia cleaning during menses	No	15	3.5
	Yes	415	96.5
Material used in cleaning genitalia	Only water	115	26.7
	Towel	7	1.6
	Soap and water	308	71.6
Overall MHM practices	Good MHM practices	365	84.9
	Poor MHM practices	65	15.1

**Table 3:** Determinants of menstrual hygiene management practices among schoolgirls in West Gonja Municipality.

Determinants	Measures	Overall MHM practice		*p* value	COR (95% CI)	AOR (95% CI)
		Good	Poor			
Age	<15 years	111 (81.6%)	25 (18.4%)	0.198	Ref^∗^	Ref^∗^
	≥15 years	254 (86.4%)	40 (13.6%)		1.4 (0.83-2.47)	1.1 (0.59-2.06)
Ethnicity	Kamara	69 (81.2%)	16 (18.8%)	0.260	Ref^∗^	
	Gonja	197 (87.6%)	28 (12.4%)		1.6 (0.83-3.20)	
	Others	99 (82.5%)	21 (17.5%)		1.1 (0.53-2.24)	
Category of school						
	Private	20 (55.6%)	16 (44.4%)	**≤0.001**	Ref^∗^	Ref^∗^
	Public	345 (87.6%)	49 (12.4%)		**5.6 (2.74-11.60)**	**6.0 (2.64-13.59)** ^∗∗∗^
Residence	Rural	171 (79.2%)	45 (20.8%)	**0.001**	Ref^∗^	Ref^∗^
	Urban	194 (90.7%)	20 (9.3%)		**2.6 (1.45-4.49)**	**2.8 (1.55-5.18)** ^∗∗^
Person living with						
	Both parents	204 (83.6%)	40(16.4%)	0.227	Ref∗	
	Mother/father only	77 (87.5%)	11 (12.5%)		1.4(0.67-2.81)	
	Relatives	77 (86.5%)	12 (13.5%)		1.3(0.63-2.52)	
	Boarding	5 (100.0%)	0 (0.0%)		1	
	Friends	2 (50.0%)	2 (50.0%)		0.2(0.03-1.43)	
Mother's job						
	Formal job	21 (77.8%)	6(22.2%)	0.287	Ref^∗^	
	Informal job	344 (85.4%)	59(14.6%)		1.7(0.65-4.30)	
Pocket money						
	No	213 (80.4%)	52(19.6%)	0.001	Ref^∗^	Ref∗
	Yes	152 (92.1%)	13(7.9%)		**2.9(1.50-5.43)**	**2.5(1.27-4.86)**∗∗
Overall knowledge						
	Sufficient	231 (84.3%)	43(15.7%)	0.658	Ref^∗^	
	Insufficient	134 (85.9%)	22(14.1%)		1.1(0.65-1.98)	

## Data Availability

All the relevant data in this study are used in this manuscript but can be made available upon request.

## Abbreviations

MHM: Menstrual hygiene management
JMP: Joint Monitoring Program
WHO: World Health Organization
JHS: Junior High School
UNICEF: United Nations International Children's Emergency Fund.

## Supplementary Materials

Supplementary Materials Supplemental Table s1 (docx.) is the knowledge on menstruation and menstrual hygiene practice scoring scale.
