# Supplementary material for: Determinants of Menstrual Hygiene Management Practices among Schoolgirls: A Cross-Sectional Study in the Savannah Region of Ghana
Source: Infect Dis Obstet Gynecol. 2022 Aug 8;2022:7007117. doi: 10.1155/2022/7007117 (PMC9377905; doi:10.1155/2022/7007117)
Supplement: Supplementary Materials — Supplemental Table s1 (docx.) is the knowledge on menstruation and menstrual hygiene practice scoring scale. [file 7007117.f1.docx]

| **KNOWLEDGE AND PRACTICE SCORING SCALE** | | |
| --- | --- | --- |
| KNOWLEDGE SCORING SCALE | | |
| Question | Option checked [✔] | Score (0 or 1) |
| What is menstruation | Natural Process (physiological) [✔]  Disease (Pathological) [✔]  Curse [✔]  Don’t know [✔] | 1  0  0  0 |
| Cause of menstruation? | Natural Process (Hormones) [✔]  Curse of god [✔]  Caused by disease [✔]  Don’t know [✔] | 1  0  0  0 |
| Source menstrual blood comes | Vagina [✔]  Bladder [✔]  Uterus [✔]  Abdomen [✔]  Don’t know [✔] | 0  0  1  0  0 |
| Heard of menstruation before menarche | Yes [✔]  No [✔] | 1  0 |
| Know about menstrual hygiene | Yes [✔]  No [✔] | 1  0 |
| Foul odor during menstruation? | Yes [✔]  No [✔] | 1  0 |
| Menstrual blood is unhygienic? | Yes [✔]  No [✔] | 1  0 |
| PRACTICES SCORING SCALE | | |
| Use of absorbent material during period or menses? | Yes [✔]  No [✔] | 1  0 |
| Which absorbent material do you use mostly during menstruation? | Sanitary pad [✔]  Tissue paper [✔]  Cotton [✔]  Cloth [✔] | 1  0  0  1 |
| Reuse absorbent materials? | Yes [✔]  No [✔] | 1  0 |
| If reused, how do you clean/wash it? | Only water [✔]  Soap and water [✔] | 0  1 |
| If reused, where do dry the absorbent material? | Sunlight [✔]  Inside the room [✔] | 1  0 |
| Frequency of absorbent material change? | Once [✔]  Twice [✔]  Thrice or more [✔] | 0  1  1 |
| Bath during menses? | Yes [✔]  No [✔] | 1  0 |
| How often do you bath during Menstruation/Period? | Daily  Twice daily  Thrice daily  More than 3 times  Until the end of the menses | 0  1  1  1  0 |
| Clean your genitalia during menstruation? | Yes [✔]  No [✔] | 1  0 |
| What do you use you to clean your genitalia (private part) during your menses? | Only water  Towel  Soap and Water | 1  0  0 |
